# Role of oxygenation devices in alleviating the oxygen crisis in India

**DOI:** 10.1371/journal.pgph.0002297

**Published:** 2023-08-17

**Authors:** Deepshikha Batheja, Vinith Kurian, Sharon Buteau, Neetha Joy, Ajay Nair

**Affiliations:** 1 One Health Trust (Formerly CDDEP), Bengaluru, Karnataka, India; 2 LEAD at Krea University, Chennai, Tamil Nadu, India; 3 ACT Grants, Bengaluru, Karnataka, India; 4 Swasth Alliance, Bengaluru, Karnataka, India; Dr. D.Y. Patil Medical College, Hospital and Research Centre, Dr. D.Y. Patil Vidyapeeth, Pune, INDIA

## Abstract

There has been an unprecedented increase in global demand for medical oxygen equipment to solve the acute oxygen shortages caused by SARS-CoV-2 infection. The study aims to assess the value of improved access and use of Oxygen Concentrators (OCs) and cylinders during the COVID-19 pandemic in India. This evaluation is relevant to strengthening health systems in many resource-constrained Low- and Middle-Income Country (LMIC) settings. Using a Probability Proportional to Size (PPS) sampling method, primary surveys were conducted in 450 health facilities across 21 states in India. The primary outcomes measured were self-reported utility of oxygenation devices in meeting the oxygen demand in the short-run and long-run utility of devices compared to the pre-oxygen-devices-distribution-period. We perform bivariate and multivariate regression analyses. Around 53–54% of surveyed facilities reported that the distributed oxygenation devices helped meet oxygen demand in the short run and are expected to increase their long-run capacity to admit non-COVID patients with oxygen needs. The timely availability of technicians was associated with meeting oxygen demand using the additional oxygenation devices at the facilities. Facilities that increased the number of staff members who were able to administer oxygen devices were at higher odds of reducing the administrative load on their staff to organize oxygen support in the long run. Hospital infrastructure was also associated with long-run outcomes. We find that oxygenation devices such as cylinders and OCs were useful in addressing the oxygen demand during the COVID-19-related oxygen emergency. Overall production of oxygen to meet the demands and investments in training biomedical engineers/technicians to administer oxygen could help save lives.

## Introduction

The COVID-19 crisis amplified a critical vulnerability in the healthcare infrastructure of many Low- and Middle-Income Countries (LMICs)—a severe shortage of medical oxygen [1–6]. Critically ill patients need medical-grade oxygen for invasive ventilation and low- and high-flow oxygen therapies [7]. An estimated half a million people in LMICs need 1.1 million cylinders of oxygen per day, with 25 countries in 2021 reporting surges in demand for medical oxygen up to a degree of shortage multiple of 60, the majority of countries with oxygen deficits are concentrated in Africa, Middle East and South Asia [6, 8]. In 2015, the Lancet Commission on Global Surgery reported that approximately one-quarter of hospitals surveyed in resource-limited countries lack sufficient oxygen supply [9, 10].

On 26th April 2021, India saw the highest daily tally of the new SARS-CoV-2 infections ever recorded at the time in the world, 360,960 cases per day, taking the pandemic total to 16 million cases, globally. Among the myriad complications associated with responding to the crisis, the demand for oxygen required for critical care patients significantly exceeded its supply. Hospitals across the country faced a major crisis of short-term, mobile sources of oxygenation, imperative to the health of affected patients, disproportionately increasing mortality rates. As a response to the crisis, the country saw an influx of oxygen cylinders, oxygen concentrators (OC) and the setting up of Pressure Swing Adsorption (PSA) plants [11, 12]. The import of oxygen therapy apparatus has increased drastically over the past three years. The first lockdown was announced in end-March 2020 and imports of oxygen apparatus stood at 1.74 million units, which is almost three times what it was in 2017–18. In 2020–21, the number further went up to 2.5 million units, according to the Directorate General of Commercial Intelligence and Statistics [13].

While medical equipment donation to low-resource settings is also a frequently used strategy to address existing disparities, there is a paucity of supportive evidence of its utility [14–16]. It is crucial to analyse the benefit of these devices and the associated costs, such as ease of use and cost of maintenance/repair before more investments are made in procuring more oxygenation devices. Some associated challenges such as infrastructure gaps, lack of technological and maintenance capabilities, and non-prioritisation of essential supplies have previously been highlighted in other developing-country contexts [3, 17–20]. We contribute to this literature by assessing the utility and barriers to access and use of OCs and cylinders in the COVID-19 pandemic in the Indian setting during a global emergency. We focus on both short-term and long-run outcomes to explore how donated medical equipment such as oxygenation devices can be used and maintained effectively to achieve a longer life. Such an evaluation is also essential to make policy-level changes, as we work towards strengthening the health system to tackle and prevent similar crises in the future in resource-constrained LMIC settings.

### Methodology

The study uses a primary cross-sectional survey data collection design from 450 healthcare facilities across 21 states in India where a philanthropic initiative by ACT Grants and Swasth Alliance had distributed over 50000 oxygen equipment during the second wave. We used a logistic regression model to study whether oxygenatation devices were valuable in COVID-19.

### Sample selection

The sampling frame consisted of all hospitals that received oxygenation devices (OCs and/or cylinders) as part of the COVID-19 relief initiatives of ACT grants and the Swasth Alliance. This includes a total of 2158 facilities. The sample selection process was conducted in two stages. First, to identify a selection of districts that represent varying levels of poverty and COVID-19 caseload intensities. Second, a stratified selection of different types of facilities that received oxygen devices were randomly selected to have representation across different types of facilities ranging from COVID Care Centres, Private facilities and Government facilities of different grades.

#### District sample selection

To generate a representative sample of districts, a combination of three secondary datasets was utilised.

1. Healthcare Facility MIS Data (To map Facilities where surveys can be conducted): This data is maintained internally by ACT Grants with information on the distribution of oxygen devices at a facility level.
2. District Level COVID-19 Caseload data (https://www.covid19india.org/): To measure COVID Intensity at a district level.
3. District Level Multi-Poverty Index Data (https://mppn.org/paises_participantes/india/): To measure the intensity of poverty among beneficiary districts.

The Probability Proportional to Size (PPS) sampling method was adopted by assigning the proportion of oxygen devices distributed within each district as the variable indicative of the size of the district, using the healthcare facility MIS data. This was done to ensure that the proportional representation of states and districts was in line with the distribution of devices within these districts. A sample of 100 districts was drawn for the survey, which accounted for 60% of the total equipment distributed. The other two datasets on district-level poverty and COVID-19 caseload were used mostly for validation of the selected sample to check whether a mix of high/low COVID Intensity districts and high/low intensity of poverty were selected. The District Level COVID-19 Caseload data was used to categorize the districts into high-COVID intensity and low-COVID intensity groups based on a median split of the ratio of the number of deceased to the number of confirmed cases (in thousands). The District Level Multi-Poverty Index was used to classify the districts into high-poverty and low-poverty categories based on a median split of the multidimensional poverty index.

#### Facility sample selection

For sampling the facilities, we relied on MIS data from ACT Grants and Sattva. A stratified random sample of 6 facilities was conducted in each of the 100 sampled districts, across the following strata of healthcare facilities where oxygen devices were sent- (i) COVID Care Centres, (ii) Government Hospitals (Above the Primary Level), (iii) Government PHC/CHC Facilities (The data was grouped for CHC and PHC facilities because both followed similar health response protocols in response to COVID-19, i.e., temporary monitoring of outpatients and transfer to the higher facility), (iv) Private Facilities, and (v) Other Facilities (example: Military/ Railway Hospitals).

A total of 558 endpoint facilities were selected for the primary data collection exercise. An oversampling of facilities was done to account for non-responses/ refusals in participating in the survey exercise among facilities. A total of 450 healthcare facilities were surveyed in person between October and December 2021. The survey was mainly carried out by an organization called LEAD by Krea University in India, with support from several grassroots-level NGOs across different states in India.

We received a list of Single Points of Contact (SPOCs) for each of these 450 selected healthcare facilities from our partner organizations in this project, Sattva and ACT Grants. The SPOCs were contacted to set up a date for the survey. On reaching the facility, the enumerators contacted the medical officer in-charge or the medical superintendent for the survey. In most facilities, these formed the respondents for the survey. However, for specific sections (eg: device storage and maintenance, patient loads), the officer in-charge would direct the enumerators to the nursing supervisor, equipment manager/ store in-charge to collect the responses.

### Outcomes measured

The survey instrument was designed using the SurveyCTO platform and administered using a tablet device in-person (See S1 Text to access the survey questionnaire). The survey instrument captured questions on the perceived impact of the supply of oxygen devices by ACT Grants and Swasth Alliance on the COVID-19 response. The following outcomes were used to measure the utility of oxygenation devices in alleviating oxygen supply shortages in the short-run and long-run:

1. Effect of additional delivery of OCs and cylinders on oxygen demand in the short-run was explored using a Likert-scale question and combining responses such as “Significantly decreased” and “Completely/fully met” oxygen demand to indicate a positive response.
2. Expected increase in capacity of the facility in the long-run was a self-reported question on whether respondents/facilities would be able to admit non-COVID patients with oxygen needs in future (long-run).
3. Expected reduced administrative load on staff to organize oxygen support in the long-run was a self-reported yes/no question on the same.

#### Ethical considerations

All respondents provided verbal informed consent to participate in the study. No personally identifiable information was used for any of the analysis. The assessments did not influence the time or place of staff responsible for care at facilities nor impose significant additional burden on patients or staff. The study protocol received approval from the Human Subjects Committee of the Institute of Financial Management and Research (IFMR IRB), Ref (PIRB00007107; FWA00014616; IORG0005894).

### Patient and public involvement statement

We piloted the survey tool among a few hospital administrators, whose inputs helped us refine the survey design.

### Data analysis

Descriptive, bivariate, and multivariate regression analyses were conducted (See S1 Data and S2 Text to access the data and codes used for the analysis). Bivariate analyses were performed to identify significant associations between the dependent and independent variables. Those independent variables significantly associated with the dependent variables at a p-value <0·05 were entered into the multivariate model. Since our main outcomes are binary, we used a logistic regression model to identify their associations with various independent variables (Odd’s ratio for bivariate analysis, adjusted Odd’s ratio for multivariate analysis). We cluster the standard errors at the state-level. Statistical analyses were performed using STATA v.16 software.

Data on the variables of interest were extracted from the datasets stored in software-encrypted files, cleaned and recoded into categorical values. Less than 5% of the data was missing. Missing data were imputed with mean responses to the question observed in the overall data.

### Definition of variables

#### Independent variables

We test for associations of dependent variables with independent variables such as the characteristics of the healthcare facilities: type of facility (where comparison/reference category is covid care centre, private and other facilities i.e., all non-government and private entities), geographic location being rural (with urban as the reference category, which includes “urban” and “mostly urban” areas), the availability of oxygenation devices (reference category is facilities that did not receive any OCs or cylinders) and the timeliness of receiving devices with respect to the second wave of COVID-19 at the district level. Self-reported average patient load during the 2nd wave (reference category is”0” patient load) was also included to measure the facility’s capacity to cater to COVID-19 patients. Variables measuring changes to healthcare personnel include the increase in medical equipment administrators and technicians during COVID-19 (reference category is facilities that did not increase the number of staff members who can administer oxygen). We also included covariates that capture the availability of infrastructure at the healthcare facility including hygiene levels (reference category is shortage of staff or equipment to maintain hygiene), storage availability (reference category is “No” or limited storage available), availability of electrical outlets (reference category is less than sufficient working electricals outlets), availability of distilled/sterile water (reference category is less than sufficient access to distilled/sterile water).

## Results

A total of 450 healthcare facilities were surveyed, comprising 22 (5%) COVID Care Centres, 157 Government Hospitals (Above the primary care level), 189 Government Primary Health Centres (PHC) / Community Health Centres (CHC) and 31 Private facilities. On average 26 oxygen devices (including OCs and Oxygen Cylinders) were delivered to Government Hospitals followed by 18.5 devices at COVID Care Centres (Table 1). Private hospitals received 12.9 devices on average while PHC/CHC level Government facilities received 5.7 devices. In terms of location, 315 (70%) of the facilities were located in rural (More than 75% rural population) or mostly rural (50–75% of the rural population) districts while the remaining 135 (30%) of facilities were from urban (More than 75% urban population) or mostly urban (50–75% urban population) districts. In terms of the timeliness of receiving devices, 318 (71%) of devices were delivered during the peak of the second wave of COVID-19 in India. To define the peak, we plotted the daily active caseload data for the entire second wave period and then identified the peak based on observations that correspond to the 75th percentile or higher in the caseload. Over 133 (85%) of Government facilities received the devices during the peak while only 100 (53%) of PHC/CHC level facilities, located mostly in rural geographies received their devices during the peak months.

**Table 1:** Healthcare facility characteristics (sample).

Variables	COVID Care Centres	Government Hospitals	Govt.–PHC/CHCs	Private Hospitals	Other Facilities	Total
**No. of Facilities (N (%))**	22 (5%)	157 (35%)	189 (42%)	31 (7%)	51 (11%)	450 (100%)
**Distribution by District Type (N (%))**						
*Rural*	9 (41%)	56 (36%)	120 (63%)	5 (16%)	6 (12%)	196 (44%)
*Mostly Rural*	4 (18%)	44 (28%)	48 (25%)	8 (26%)	15 (29%)	119 (26%)
*Urban*	5 (23%)	22 (14%)	14 (7%)	15 (48%)	17 (33%)	73 (16%)
*Mostly Urban*	4 (18%)	35 (22%)	7 (4%)	3 (10%)	13 (25%)	62 (14%)
**Oxygenation Devices Delivered by ACT Grants & Swasth Alliance (N)**						
*No*. *of OCs Delivered (Mean)*	17.2	19.7	4.2	10.2	7.7	
*No*. *of OCs Delivered (Median)*	10	8	3	2	5	4.5
*No*. *of Cylinders Delivered (Mean)*	1.2	6.3	1.5	2.6	1.0	
*No*. *of Cylinders Delivered (Median)*	0	0	0	0	0	0
*No*. *of Total Devices Delivered (Mean)*	18.5	26.0	5.7	12.9	8.8	
*No*. *of Total Devices Delivered (Median)*	11	15	5	2	5	
**Timing of Receiving Oxygen Devices (N (%))**						
*During the Peak of the Second Wave*	18 (82%)	133 (85%)	100 (53%)	26 (84%)	41 (80%)	318 (71%)
*After the Peak of the Second Wave*	2 (9%)	20 (13%)	86 (46%)	4 (13%)	9 (18%)	121 (27%)
**No. of patients requiring oxygen support (Daily Mean)**						
*Before the Peak of the Second Wave*	12.9	39.9	5.2	24.6	13.4	19.9
*During the Peak of the Second Wave*	48.5	83.6	10.2	43.1	24.8	41.3
**Healthcare Infrastructure Availability**						
*Access to adequate electrical capacity*	20 (91%)	138 (88%)	150 (79%)	30 (97%)	47 (92%)	385 (86%)
*Adequate levels of hygiene & sanitation*	20 (91%)	116 (74%)	140 (74%)	29 (94%)	45 (88%)	350 (78%)
*Availability of storage facility for equipment*	18 (82%)	118 (75%)	141 (75%)	30 (97%)	46 (90%)	353 (78%)
*Adequate quality of water available*	7 (32%)	91 (58%)	70 (37%)	22 (71%)	28 (55%)	218 (48%)
*Facilities with increase in number of staff being able to administer oxygen support to patients*	13 (59%)	98 (62%)	88 (47%)	24 (77%)	27 (53%)	250 (56%)

On average, the daily number of patients requiring oxygen support was 19.9. This is more than doubled to 41.3 patients daily during the peak months of the second wave in India. At Government facilities, the requirement for oxygen support nearly doubled from 39.9 patients before the second wave to 83.6 patients during the peak months of the second wave. Government hospitals reported being more affected by the increased demand for oxygen support compared to other types of healthcare facilities.

### Facility infrastructure

Table 1 describes the infrastructure available at the surveyed healthcare facilities and the challenges to the long-term usage of oxygenation devices received by them. In terms of the availability of adequate electrical infrastructure (in terms of functional electric outlets and uninterrupted power supply), 350 (86%) of facilities reported the positive. Lack of distilled water for the oxygen concentrator was a bottleneck reported by over half 232 (52%) of facilities with COVID Care Centres and PHC/ CHC level facilities faring worse. 210 (17%) of facilities reported that there was an adequate availability of medical equipment repair technicians in case of any issues with the devices they received. While 24 (77%) of private facilities reported adequacy, only 70 (37%) of PHC/ CHC level facilities, located largely in rural or mostly rural districts had access to a sufficient number of technicians.

While private facilities 29 (94%) and COVID Care Centres 20 (91%) reported adequate levels of hygiene and sanitation, over one-fourth of Government facilities both at secondary and tertiary levels 116 (74%) and at the PHC/ CHC levels report not being able to maintain adequate levels of hygiene and sanitation at the facility. In a similar vein, while private facilities 30 (97%) and COVID Care Centres 15 (68%) reported adequate storage facilities for oxygenation equipment while over one of Government Hospitals lacked sufficient storage facilities with implications for long-term usage of these devices. Over 250 (56%) of facilities also reported that there has been an increase in the number of staff being able to administer oxygen support to patients since the beginning of the second wave.

### Utility of oxygen equipment in alleviating supply shortages in the short-run and long-run

#### Effect of additional delivery of OCs and cylinders on oxygen demand in the short run

**Fig 1:**
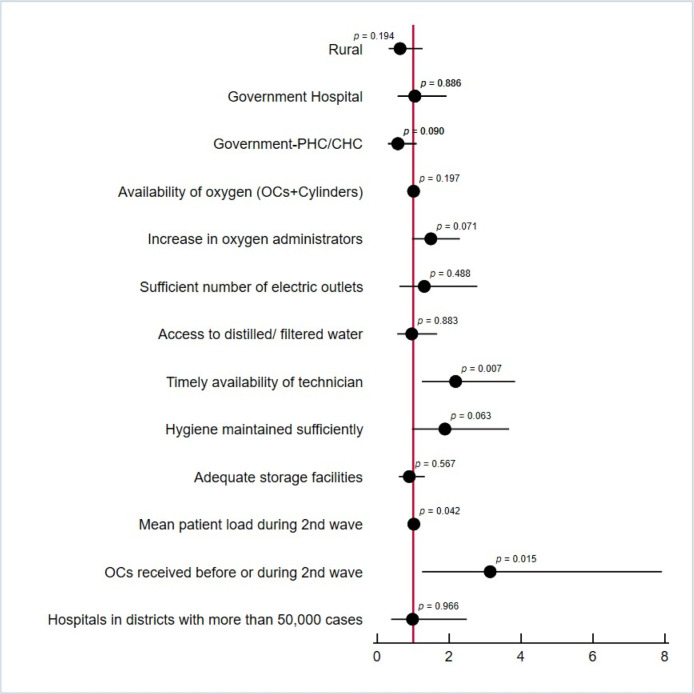
Bivariate analysis for additional OCs and cylinders on oxygen demand in the short-run.

A majority of (54%) facilities reported that the distribution of oxygenation devices helped meet their oxygen demand (Table 2). 67% of the surveyed private hospitals and 64% of covid care centers reported having benefitted from receiving these oxygenation devices in the short run in meeting the oxygen demand. The bivariate analysis associations showed that the timely availability of technicians, the average load of COVID-19 patients during the second wave and the timely availability of oxygenation devices such as OCs were predictors of additional oxygenation devices having a significant impact on meeting the oxygen demand at the facility (Fig 1). The multivariate analysis confirmed that facilities with adequate availability of medical equipment repair technicians (AOR: 1.77, CI: 1.02–3.08, p-value: 0.04) and those receiving oxygenation devices before the start of the second wave (AOR: 2.60, CI: 1.04–6.49, p-value: 0.04) were at higher odds of meeting the oxygen demand at the facility with the delivery of additional oxygenation devices.

**Table 2:** Utility of devices in alleviating oxygen supply shortages.

Variables	COVID Care Centres	Government Hospitals	Government–PHC/CHCs	Private Hospitals	Other Facilities	Total
**Number of facilities**	22 (5%)	157 (35%)	189 (42%)	31 (7%)	51 (11%)	450 (100%)
**Increased capacity to admit non-COVID patients with oxygen needs in future (N (%))**						
*After the Second Wave (Long-Run)*	9 (41%)	91 (58%)	107 (57%)	14 (45%)	18 (35%)	239 (53%)
**Reduced administrative load on staff to organize oxygen support (N (%))**						
*After the Second Wave (Long-Run)*	6 (27%)	35 (22%)	45 (24%)	13 (42%)	9 (18%)	108 (24%)
**Effect of additional delivery of devices on oxygen demand (short-run) (N (%))**						
*Significantly helped* or fully met oxygen demand	14 (64%)	85 (55%)	96 (50%)	21 (67%)	26 (51%)	242 (54%)

#### Expected increase in capacity of the facility in the long run

**Fig 2:**
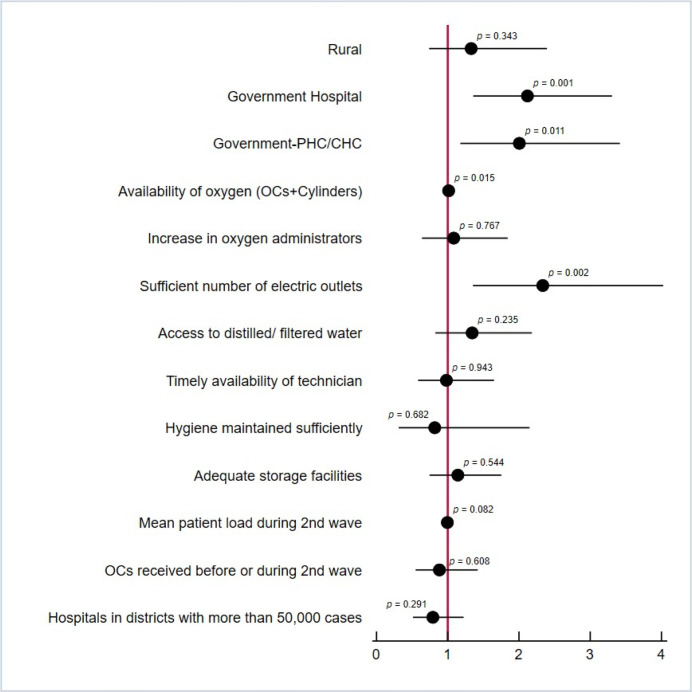
Bivariate analysis for additional OCs and cylinders on expected increase in capacity of the facility in the long-run.

Around 239 (53%) facilities expected an increase in the capacity of the facility to admit patients with non-COVID illnesses (that require oxygen support) in the long run. Perceived capacity to admit patients with non-COVID-19 illnesses, in the long run, was highest in government hospitals (58%) and government PHCs/CHCs (57%), compared to other facilities (Table 2). As per the bivariate (Fig 2) and the multivariate analyses, the potential predictors of the expected increase in the capacity of the facility to admit non-COVID-19 patients that required oxygen in the long run included type of facility such as government hospitals (AOR:1.95, CI: 1.22–3.11, p-value: 0.005) and government PHC/CHC (AOR: 2.58, CI: 1.44–4.63, p-value: 0.001), availability of oxygenation devices (AOR: 1.01, CI: 1.00–1.02, p-value:0.015) and sufficient number of electric outlets (AOR: 2.64, CI: 1.46–4.80, p-value: 0.001).

#### Expected reduced administrative load on staff to organize oxygen support in the long-run

**Fig 3:**
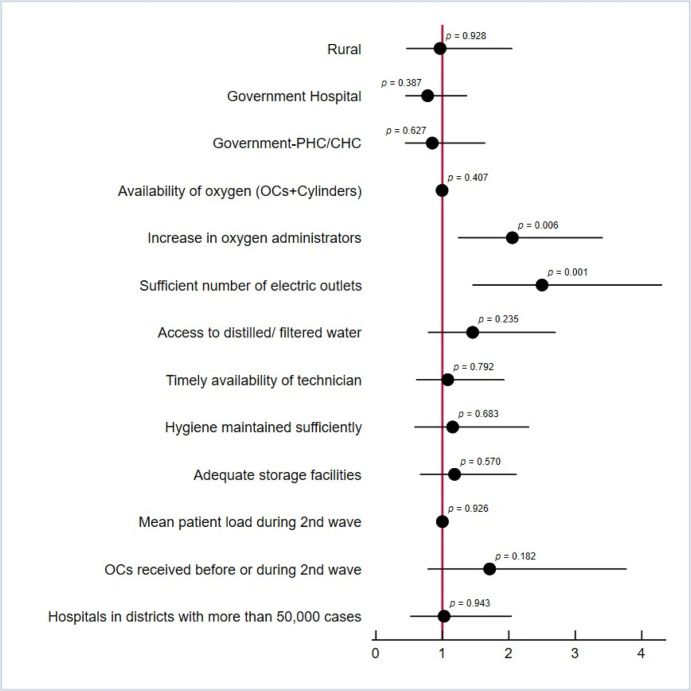
Bivariate analysis for additional OCs and cylinders on expected reduced administrative load on staff to organize oxygen support in the long-run.

In terms of reducing the administrative work for staff to organize oxygen support, 42% of the surveyed private hospitals perceive a higher impact of oxygenation devices in the long-run, compared to 24% of the overall surveyed facilities (Table 2). The bivariate and multivariate analyses showed that facilities with sufficient number of electric outlets (AOR:2.44, CI: 1.34–4.45, p-value: 0.003) and facilities that increased the number of staff members that were able to administer oxygenation devices at the beginning of the second wave (AOR: 2.02, CI: 1.21–3.37, p-value: 0.007) were at higher odds of expecting a reduction in the administrative load on their staff to organize oxygen support in the long run (Fig 3).

## Discussion

The acute oxygen crisis experienced during the COVID-19 pandemic has necessitated comprehensive efforts to manage oxygen shortages [14, 15, 21]. This paper explores the benefits of increased availability and utilization of Oxygen Concentrators (OCs) and cylinders during the COVID-19 pandemic in India. The study findings underscore the importance of not only increasing oxygen availability but also ensuring sustainable and efficient usage at healthcare facilities. A majority of facilities (54%) reported that the distribution of oxygenation devices helped meet their oxygen demand during the peak months of the second COVID-19 wave in India. Facility-level factors such as trained manpower and infrastructural sufficiency were identified as key determinants of meeting oxygen demand.

Timeliness in receiving oxygen devices before or during the onset of the surge in demand enabled facilities to better allocate and efficiently use the available oxygen for COVID patient caseloads. Availability of oxygen also led to an increase in the ability to treat non-COVID-related patients, especially in government facilities and PHC/ CHC level facilities facing resource constraints. Making oxygen devices available in government facilities is crucial in developing an inclusive healthcare system as the poorest quintile of the population has a higher dependency on public healthcare facilities for in-patient support compared to other income classes, according to the [22].

The paper highlights that investing in training personnel to administer and maintain oxygen can provide efficiency gains in the longer term and reduce administrative burden. Our findings also suggest that facilities that were able to increase the number of staff able to administer oxygen devices were more likely to be able to meet their oxygen needs. Long-term maintenance of devices with the timely availability of biomedical repair technicians is an important predictor of facilities being able to meet their oxygen demand. The findings of this study also have wide policy implications for employment generation in the healthcare sector. For instance, there could be a potential collaboration with the National Skill Development Corporation in the Indian setting to create skill-based employment for the administration and repair of oxygenation devices.

While investing in personnel may take years to bear fruition, government agencies and private entities donating or subsidising these devices must carefully consider long-term maintenance budgets when assessing the feasibility and impact of their contributions, particularly regarding the ongoing cost burden of related infrastructural facilities [23]. These include funds to ensure maintenance of adequate electrical load and outlets which are important for the operation of oxygen concentrators in particular. In rural hospitals that experience frequent power outages and have limited access to electrical outlets, alternative and sustainable solutions, such as the installation of solar panels, could potentially address this issue [24]. By harnessing solar energy, healthcare facilities can ensure a more reliable and continuous power supply, thereby supporting the uninterrupted operation of essential medical equipment. Adoption of innovative solutions can improve the resilience of healthcare facilities to power outages and ensure the provision of quality healthcare services in resource-constrained settings. Furthermore, the use of solar panels aligns with the broader goal of promoting sustainable and environmentally conscious practices within the healthcare sector.

Another issue in the effective utilization of oxygenation devices is that the maintenance of equipment is significantly influenced by cultural and bureaucratic factors in the public healthcare domain [25–27]. Under-reporting of breakdowns is common due to the absence of maintenance funds and repair technicians. Creating an enabling environment is crucial to improve device lifetime and address associated cost burdens.

Overall, efforts to enhance oxygen availability and sustainable usage in healthcare facilities require a multifaceted approach. This study emphasizes the significance of trained manpower, timely device allocation, long-term maintenance, and the consideration of cultural and bureaucratic factors. Creative solutions, such as the implementation of barcodes or QR codes on oxygenation devices can be beneficial in significantly improving asset management and facilitation of tracking, monitoring, repairing, and replacement of these devices [28]. By assigning unique codes to each device, healthcare facilities can easily identify and keep a record of their inventory, ensuring efficient management of these life-saving devices. The utilization of barcodes or QR codes also enables better redistribution of oxygenation devices based on specific needs, leading to their maximum utilization [28]. For instance, during the post-pandemic period, primary healthcare centers (PHCs) and community health centers (CHCs) that had increased their capacity and number of beds to address the COVID-19 surge may find that the oxygenation devices they received are not fully utilized. In such cases, the barcode or QR code system can facilitate the redistribution of these devices to other Government settings, such as old-age care homes with older residents who may have sustained high oxygen requirements even in the post-pandemic period.

Another important issue is that sourcing oxygen equipment remains problematic, as they are primarily produced in developed, high-income countries. Even devices designed for low-resource settings may lack operating manuals in the local vernacular, hindering proper usage and maintenance. However, facility-specific challenges limit the generalizability of specific solutions.

In the context of limited resources and technical constraints, concentrators have emerged as a user-friendly option for home-based oxygen therapy, particularly in low- and middle-income countries (LMICs). These devices are designed to be easily operated without requiring extensive technical expertise [29]. However, to ensure the effective utilization of oxygen concentrators, policymakers can take certain measures. One important step is the development of manuals or instructional videos in local languages, targeting both operators and users of oxygen concentrators at the hospital and patient level. These materials should be tailored to the specific context and resources available in LMIC settings. By providing instructions in local languages, the usability and accessibility of the devices can be significantly enhanced, promoting their effective utilization. Additionally, these manuals should include comprehensive information that may not be readily available on the internet in local languages. For instance, guidelines on the expiry date of oxygen-filled cylinders are essential to ensure the safety and efficacy of the therapy. Including such critical information in the manuals can address potential gaps in knowledge and help users make informed decisions regarding the use and maintenance of the concentrators.

To boost the availability of last-mile oxygen delivery and the long-term use of oxygen cylinders, oxygen banks at the district level can be established. This approach would facilitate efficient oxygen distribution and mitigate future shortages [30]. By implementing these strategies, policymakers can enhance the usability and effectiveness of oxygenation devices in LMIC settings, ultimately improving access to oxygen therapy and supporting better patient outcomes.

## Conclusion

We carried out a survey in health facilities which received oxygenation devices during the second wave of COVID-19 in India on the reported short-run and expected long-run utility of these devices. Our findings suggest that these devices helped meet the short-run oxygen demand and are likely to increase the hospital capacity in the long-run to admit non-COVID patients. However, these devices can be fully utilized to meet both short and long-run oxygen shortages only if there are skilled technicians and oxygen administrators to help in the use and repair of these devices. It will also require the creation of supportive infrastructure such as sufficient number of electrical outlets in health facilities, which is a particularly relevant result for the resource-constrained LMIC setting.

### Limitations of the study

A retrospective assessment limits the ability to determine cause and effect which can attribute impact to a particular intervention. There was also limited flexibility in the selection of the sample for the primary endpoint facilities survey with a focus on states that received greater support in terms of equipment allocation and distribution. This has implications for the generalizability of certain results identified under the intervention. There was insufficient information on the total number of OCs and cylinders present at a facility to permit a baseline assessment. The survey tool focuses on the combined effect of OCs and cylinders. The two devices are likely to have varied long-term use cases. Further, we did not look at the characteristics of oxygen devices such as the brand, as this was not the objective of the study. In addition, sufficient time had not passed to assess the durability of the oxygenation devices.

We are also unable to differentiate between PHC and CHC facilities. This is because for sampling the facilities, we relied on health care MIS data internally maintained by the NGO- ACT Grants. The data was grouped for CHC and PHC facilities in the MIS data. However, the inability to distinguish between CHC and PHC is not concerning for this study. This is because both facilities have similar infrastructural capacities (eg: number of hospital beds) and followed similar health response protocols in response to COVID-19 (i.e., temporary monitoring of outpatients and transfer to higher facility).

## Supporting information

S1 Text This PDF file contains the survey questionnaire used in this paper. (DOCX)

S2 Text This file contains the codes that were used to analyze the data. (DOCX)

S1 Data This file contains de-identified and anonymized healthcare facility-level raw primary data used in the analysis. (XLSX)
