# Supplementary material for: Role of oxygenation devices in alleviating the oxygen crisis in India
Source: PLOS Glob Public Health. 2023 Aug 17;3(8):e0002297. doi: 10.1371/journal.pgph.0002297 (PMC10434891; doi:10.1371/journal.pgph.0002297)
Supplement: S1 Text — (DOCX) [file pgph.0002297.s001.docx]

| **Endpoint Facilities Survey** | | | | | | |
| --- | --- | --- | --- | --- | --- | --- |
| Q. No. | Question | Response Code | Response Type | Instructions to Programmer |  |  |
|  | **SECTION O: Background Information** | | | | |  |
| O1 | ID (Internally Generated) |  |  |  |  |  |
| O2 | State | prefill |  | Prefilled |  |  |
| O3 | District | prefill |  | Prefilled |  |  |
| O4 | Address | prefill |  | Prefilled |  |  |
| O5 | Hospital Name | prefill |  | Prefilled |  |  |
| O6 | Type of End Facility |  |  |  |  |  |
| O7 | Respondent Title |  |  |  |  |  |
| O8 | Contact Number |  |  |  |  |  |
| O9 | email id |  |  |  |  |  |
| **SECTION A: Oxygen Concentrators and Cylinders** | | | | |  |  |
| A1 | When did you receive all or most of the oxygen concentrators and cylinders with respect to the onset of the second wave in your local area? | 1 2 3 | Before the peak of the second wave During the peak of the second wave After the peak of the second wave | Select One |  |  |
| A2 | Before the second wave of the pandemic, what was the average daily number of patients requiring oxygen support that could be admitted in your facility? |  | ______________ | Enter Number |  |  |
| A3 | During the most difficult months of the pandemic, what was the average daily number of patients requiring oxygen support that sought admission in your facility? |  | ______________ | Enter Number |  |  |
| A4 | To what extent did the delivery of additional oxygen concentrators and cylinders help meet the demand for oxygen in your facility | 1 2 3 4 | Not at all Moderately Significantly Completely | Select One |  |  |
| A5 | At present, what is the average number of patients requiring oxygen support that visit your facility per day? |  | ______________ | Enter Number |  |  |
| A6 | At present, what proportion of these patients were admitted for COVID-related illnesses? |  | ______________ | Enter Number (0-100) |  |  |
| A7 | How many staff members at your facility that are able to administer the use of the oxygen concentrators and cylinders to patients ? |  | ______________ | Enter Number |  |  |
| A8 | Did the number of staff members at your facility that are able to administer the use of the oxygen concentrators and cylinders to patients increase from the beginning of the second wave? | 1 2 | Yes No | Select One |  |  |
| A9 | How did the supply of oxygen devices by ACT/ Swasth impact your COVID-19 response? Select all that apply | 1  2  3  4  0 -888 | Able to accept more patients needing oxygen support Reduced need of transferring patients to other healthcare facilities Reduced instances of severe COVID patients Reduced administrative work for staff to organize oxygen support Did not impact in anyway Others, Specify ________ | Select Multiple |  |  |
| A10 | In your estimation, what is the total no. of COVID patients that you were able to treat during the second wave of the pandemic? |  | Quantity_______ | Enter Number |  |  |
| A11 | What is the number of patients that you could have admitted without the additional supply of oxygen concentrators and cylinders? |  | Quantity_______ | Enter Number |  |  |
| **Section B: Hospital Infrastructure** | | | | | |  |
| B1 | Does your facility have an intensive care unit (ICU)? | 1 2 | Yes No |  |  |  |
| B2 | Does your facility have adequate and functioning electricity outlets to operate oxygenation devices such as Oxygen Concentrators and BiPAPs | 1  2   3 | Most electricity outlets do not work or are not adequate for devices to work Some electricity outlets work but are not adequate for the number of devices at the facility The facility has sufficient number of functional electricity outlets to meet its needs | Single Choice |  |  |
| B3 | Which of the following would describe the quality of water that is available to operate these oxygenation devices? | 1 2  3 | Access only to tap and filtered water Insufficient access to distilled / sterile water Sufficient access to distilled / sterile water | Single Choice |  |  |
| B4 | Is there adequate availability of medical equipment repair technicians to service any faults with any machines/ devices at your facility? | 1 2  3 | No technicians are available Technicians are available but significant time delays faced in access Sufficient number of technicians available in a timely manner | Single Choice |  |  |
|  |  |  |  |  |  |  |
|  |  |  |  |  |  |  |
| B5 | Are the resources available to you adequate in maintaining a good level of sanitization and hygiene at the facility? (Select all that apply) | 1  2  3 | There is a shortage of staff to maintain hygiene and sanitation There is a shortage of sanitization equipment at the facility The resources at the facility are adequate to maintain sanitization and hygiene | Multiple Choice |  |  |
| B6 | Do you have adequate storage facilities to keep medical equipment at your facility when they are not in use? | 1 2 3 | No storage facilities are available Storage facilities are available but not sufficient for all devices Storage facilities are available and sufficient | Single Choice |  |  |
| B7 | Which of the following would you say are the three most pressing concerns around hospital infrastructure that you would like to address at present? | 1  2 3  4  5  6 -888 | Access to electricity and functional electrical outlets Low quality of water available Inadequacy of medical equipment repair technicians Lack of resources to maintain sanitization and hygiene Lack of storage facilities for medical equipment No issues at present Others, Specify | Select Up to 3 |  |  |
| **Section C: Long Term Usage** | | | | | |  |
| C1 | Has your facility's dependence on sourcing external sources of oxygenation for patients changed with the additional devices received in the past 4 months (beginning of the second wave)? | 1  2  3  4 | Not Changed at All  Somewhat Decreased  Significantly Decreased  Facility requirements completely met | Single Choice |  |  |
| C2 | What impact do you see in the long term with the additional oxygenation devices that are available at the facility now? (Select All that Apply) | 1  2  3   4  5 -888 | Able to accept more patients needing oxygen support Reduced need of transferring patients to other healthcare facilities Increased capacity to admit patients with non-COVID illnesses that require oxygen support Reduced administrative work for staff to organize oxygen support Did not impact in anyway Others, Specify ________ | Multiple Choice |  |  |
| C3 | If the additional oxygen capacity you now have were to be utilized fully, how many additional patients your hospital will be able to serve per day? |  | ___________________ | Enter Number |  |  |
| C4 | Do you still require any additional oxygen infrastructure? | 1 2 | Yes No | Single Choice |  |  |
| C5 | If yes, what is the additional requirement for oxygenation that your facility requires? |  | ___________________ | Text Box Skip if D4 is 'No' |  |  |
| C6 | How do you foresee the use of the additional cylinders/ concentrators in the long term? |  | ___________________ | Qualitative (Text Box) |  |  |
| C7 | Do you anticipate any of the following challenges in using the additional oxygenation devices in the long term? You may select all that apply. | 1  2  3  4  5  6  -888 | Shortage of staff in administering these devices Lack of trained staff in handling the devices appropriately Inadequate storage facilities to keep the devices when not in use.  Increased cost associated with maintaining devices  Low demand for oxygenation devices for non-COVID illnesses  No Challenges at present  Others, Specify _____ | Multiple Choice |  |  |
