# Supplementary material for: Role of oxygenation devices in alleviating the oxygen crisis in India
Source: PLOS Glob Public Health. 2023 Aug 17;3(8):e0002297. doi: 10.1371/journal.pgph.0002297 (PMC10434891; doi:10.1371/journal.pgph.0002297)
Supplement: S2 Text — (DOCX) [file pgph.0002297.s002.docx]

use "$Data\Endpoint Facilities Survey_28 Feb 2022_final_450_additional variables.dta", clear

// A1, A5, A2, A4, C3, C4, C5, A8, A9, C1, C2, A10, A11, A12, C6, A8, A6, A7

// Questions with multiple response dummies - A10, C4, C6

// Demographic characteristic variables - ID, state, district, facility type, number of OCs delivered, number of cylinders delivered

encode o2, gen(state)

bys state: gen N=_n

bys state: gen No=_N

gen weight=1/No

// OCs delivered

tab oc

sum oc, de

gen belowaverageOCs=1 if oc<r(mean)

replace belowaverageOCs=0 if oc>=r(mean)

sum oc, de

gen belowmedianOCs=1 if oc<r(p50)

replace belowmedianOCs=0 if oc>=r(p50)

//Cylinders delivered

sum cylinder, de

gen belowaveragecylinders=1 if cylinder<r(mean)

replace belowaveragecylinders=0 if cylinder>=r(mean)

// A1 & A5

gen oxygen_demand_met=1 if a5>2

replace oxygen_demand_met=0 if a5<=2

gen peak_second_wave=(a1==2)

gen time_oc_received=(a1==1|a1==2)

//A2 & A4

su a2, de

gen ba_patient_prewave=1 if a2<r(mean)

replace ba_patient_prewave=0 if a2>=r(mean)

su a4, de

gen ba_patient_duringwave=1 if a4<r(mean)

replace ba_patient_duringwave=0 if a4>=r(mean)

bys state: egen mean_patient_prewave=mean(a2)

bys state: egen mean_patient_duringwave=mean(a4)

bys state: egen mean_patient_currently=mean(a6)

label var mean_patient_duringwave "Mean patient load during 2nd wave"

label var mean_patient_prewave "Mean patient load before 2nd wave"

label var mean_patient_currently "Mean patient load after 2nd wave (currently)"

//graph 1

preserve

collapse mean_patient_duringwave mean_patient_prewave, by(state)

twoway (line mean_patient_duringwave state) (line mean_patient_prewave state)

restore

preserve

collapse mean_patient_duringwave mean_patient_prewave mean_patient_currently, by(state)

twoway (line mean_patient_duringwave state) (line mean_patient_prewave state) (line mean_patient_currently state)

restore

// C3, C4, C5

tab c3

gen technician_available_timely=(c3==3)

tab c4

gen sufficient_hygiene_maintenance=(c4=="3")

tab c5

gen adequate_storage_facilities=(c5==3)

*Creating usability index such that:

*Index is highest when all three i.e. c3, c4 and c5 are equal to 3

egen usability=rowtotal(technician_available_timely sufficient_hygiene_maintenance adequate_storage_facilities)

tab usability o6

tab state, sum(usability)

egen norm_usability=std(usability)

egen total_usability_norm=rowtotal(norm_usability)

egen usability_index=std(total_usability_norm)

// When did they receive the OC

tab a1

tab a1, nol

gen received_OC=(a1==1|a1==2)

tab a4

gen utility_oc_2ndwave=oc/a4

gen oxygen=oc+cylinder

gen share_oc=oc/oxygen

sum share_oc, de

gen utility_oxygen_2ndwave=oxygen/a4

gen utility_oc_postwave=oc/a6

gen utility_oxygen_postwave=oxygen/a6

gen surplus_oxygen=1 if utility_oxygen_2ndwave>=1

replace surplus_oxygen=0 if utility_oxygen_2ndwave<1

encode o6, gen(facility_type)

gen govt_hospital=(facility_type==2)

gen phc_chc=(facility_type==3)

sum a8, de

gen belowaverage_technicians=1 if a8<r(mean)

replace belowaverage_technicians=0 if a8>=r(mean)

sum a11, de

gen belowaverage_treat_patients=1 if a11<r(mean)

replace belowaverage_treat_patients=0 if a11>=r(mean)

sum a12, de

gen belowaverage_admit_patients=1 if a11<r(mean)

replace belowaverage_admit_patients=0 if a11>=r(mean)

label var belowaverage_treat_patients "Patients treated during second wave of COVID"

label var belowaverage_admit_patients "Patients that could be admitted without additional oxygen"

tab a10

gen Facility=1 if facility_type==2

replace Facility=2 if facility_type==3

replace Facility=0 if facility_type!=. & Facility==.

label def facility 0 "Other types" 1 "Government Hospital" 2 "Government-PHC/CHC"

label val Facility facility

gen workload_oxygen_admin=oxygen/a8

****************************************************************

gen oxygen_demand_met_1=1 if a5>3

replace oxygen_demand_met_1=0 if a5<=3

//C1 and C2

tab c2

gen water_available=(c2==3)

tab c1

gen electricity_available=(c1==3)

//Bivariate graphs

// Outcomes - a10, a5 (oxygen_demand_met), d2, surplus_oxygen - divide this by usability and utility

//Impact of OCs and cylinders on covid response (Short-run)

preserve

ren technician_available_timely C3

ren sufficient_hygiene_maintenance C4

ren adequate_storage_facilities C5

gen A9= a9

label var A9 "Increase in oxygen administrators"

label var C3 "Timely availability of technician"

label var C4 "Hygiene maintained sufficiently"

label var C5 "Adequate storage facilities"

label var oxygen "Availability of oxygen (OCs+Cylinders)"

label var received_OC "OCs received before or during 2nd wave"

label var district_cases "Hospitals in districts with more than 50,000 cases"

label var water_available "Access to distilled/ filtered water"

label var electricity_available "Sufficient number of electric outlets"

foreach var in Rural oxygen A9 C3 C4 C5 state mean_patient_duringwave received_OC district_cases water_available electricity_available{

qui eststo `var': logistic a10_2 `var' , vce(cluster state)

}

qui eststo Facility: logistic a10_2 i.Facility, vce(cluster state)

coefplot (Rural\Facility\oxygen\A9\electricity_available\water_available\C3\C4\C5\mean_patient_duringwave\received_OC\district_cases, label(bivariate)), drop(_cons) yscale(alternate) eform xline(1) mlabposition(1) mlabgap(*2) mlabel("{it:p} = " + string(@pval,"%9.3f")) name(a10_2)

restore

//Impact of OCs and cylinders on covid response (Long-run)

preserve

ren technician_available_timely C3

ren sufficient_hygiene_maintenance C4

ren adequate_storage_facilities C5

gen A9= a9

label var A9 "Increase in oxygen administrators"

label var C3 "Timely availability of technician"

label var C4 "Hygiene maintained sufficiently"

label var C5 "Adequate storage facilities"

label var oxygen "Availability of oxygen (OCs+Cylinders)"

label var received_OC "OCs received before or during 2nd wave"

label var district_cases "Hospitals in districts with more than 50,000 cases"

label var water_available "Access to distilled/ filtered water"

label var electricity_available "Sufficient number of electric outlets"

forval i=3/4{

foreach var in Rural oxygen A9 C3 C4 C5 state mean_patient_duringwave received_OC district_cases water_available electricity_available{

qui eststo `var': logistic d2_`i' `var', vce(cluster state)

}

qui eststo Facility: logistic d2_`i' i.Facility, vce(cluster state)

coefplot (Rural\Facility\oxygen\A9\electricity_available\water_available\C3\C4\C5\mean_patient_duringwave\received_OC\district_cases, label(bivariate)), drop(_cons) eform xline(1) mlabposition(1) mlabgap(*2) mlabel("{it:p} = " + string(@pval,"%9.3f")) yscale(alternate) name(d2_1_`i')

}

restore

//Oxygen demand met

preserve

ren technician_available_timely C3

ren sufficient_hygiene_maintenance C4

ren adequate_storage_facilities C5

gen A9= a9

label var A9 "Increase in oxygen administrators"

label var C3 "Timely availability of technician"

label var C4 "Hygiene maintained sufficiently"

label var C5 "Adequate storage facilities"

label var oxygen "Availability of oxygen (OCs+Cylinders)"

label var received_OC "OCs received before or during 2nd wave"

label var district_cases "Hospitals in districts with more than 50,000 cases"

label var water_available "Access to distilled/ filtered water"

label var electricity_available "Sufficient number of electric outlets"

foreach var in Rural oxygen A9 C3 C4 C5 state mean_patient_duringwave received_OC district_cases water_available electricity_available{

qui eststo `var': logistic oxygen_demand_met_1 `var', vce(cluster state)

}

qui eststo Facility: logistic oxygen_demand_met_1 i.Facility, vce(cluster state)

coefplot (Rural\Facility\oxygen\A9\\electricity_available\water_available\C3\C4\C5\mean_patient_duringwave\received_OC\district_cases, label(bivariate)), drop(_cons) eform xline(1) mlabposition(1) mlabgap(*2) mlabel("{it:p} = " + string(@pval,"%9.3f")) yscale(alternate) name(oxygen_demand_2)

restore

//Multivariate with all variables

//oxygen demand

preserve

ren technician_available_timely C3

ren sufficient_hygiene_maintenance C4

ren adequate_storage_facilities C5

gen A9= a9

label var A9 "Increase in oxygen administrators"

label var C3 "Timely availability of technician"

label var C4 "Hygiene maintained sufficiently"

label var C5 "Adequate storage facilities"

label var oxygen "Availability of oxygen (OCs+Cylinders)"

label var bed_count "Hospitals with more than 20 beds"

label var district_cases "Hospitals in districts with more than 50,000 cases"

logistic oxygen_demand_met C3 mean_patient_duringwave received_OC, vce(cluster state)

restore

// a10 variable

preserve

ren technician_available_timely C3

ren sufficient_hygiene_maintenance C4

ren adequate_storage_facilities C5

gen A9= a9

label var A9 "Increase in oxygen administrators"

label var C3 "Timely availability of technician"

label var C4 "Hygiene maintained sufficiently"

label var C5 "Adequate storage facilities"

label var oxygen "Availability of oxygen (OCs+Cylinders)"

label var received_OC "OCs received before or during 2nd wave"

label var bed_count "Hospitals with more than 20 beds"

label var district_cases "Hospitals in districts with more than 50,000 cases"

logistic a10_2 electricity_available oxygen, vce(cluster state)

restore

// d2 variables

preserve

ren technician_available_timely C3

ren sufficient_hygiene_maintenance C4

ren adequate_storage_facilities C5

gen A9= a9

label var A9 "Increase in oxygen administrators"

label var C3 "Timely availability of technician"

label var C4 "Hygiene maintained sufficiently"

label var C5 "Adequate storage facilities"

label var oxygen "Availability of oxygen (OCs+Cylinders)"

label var received_OC "OCs received before or during 2nd wave"

label var bed_count "Hospitals with more than 20 beds"

label var district_cases "Hospitals in districts with more than 50,000 cases"

logistic d2_3 i.Facility electricity_available oxygen d7_1 d7_2 d7_3 d7_4 d7_5 d7_0, vce(cluster state)

logistic d2_4 electricity_available a9 d7_1 d7_2 d7_3 d7_4 d7_5 d7_0, vce(cluster state)

restore

*****

logistic oxygen_demand_met Rural oxygen A9 C3 C4 C5 state mean_patient_duringwave received_OC district_cases water_available electricity_available

logistic oxygen_demand_met C3 mean_patient_duringwave received_OC, vce(cluster state)
